# Using cycling as transportation and related adverse events during pregnancy: An anonymous questionnaire survey in urban Japan

**DOI:** 10.1371/journal.pone.0358856

**Published:** 2026-09-22

**Authors:** Mamoru Morikawa, Yuki Morikawa, Shintaro Yasuda, Shuji Tei, Ichiro Orino, Hikari Akise, Kozo Ashihara, Takeshi Inoue, Tadashi Okazaki, Goichi Iito, Kohei Osamura, Yasuhisa Fujiwara, Shun-ichiro Nakanishi, Takao Kamiya, Tomoo Yoshimura

**Affiliations:** 1 Department of Obstetrics and Gynecology, Kansai Medical University, Hirakata City, Osaka, Japan; 2 Graduate School of Health and Welfare Sciences, International University of Health and Welfare, Tokyo, Japan; 3 Department of Obstetrics and Gynecology, Narimoto Lady’s Hospital, Hirakata City, Osaka, Japan; 4 Department of Obstetrics and Gynecology, Fujimoto Hospital, Neyagawa City, Osaka, Japan; 5 Department of Obstetrics and Gynecology, Orino Obstetrics and Gynecology Clinic, Hirakata City, Osaka, Japan; 6 Department of Obstetrics and Gynecology, Akise Women’s Clinic, Hirakata City, Osaka, Japan; 7 Department of Obstetrics and Gynecology, Ashihara Obstetrics and Gynecology Clinic, Hirakata City, Osaka, Japan; 8 Department of Obstetrics and Gynecology, Inoue Obstetrics and Gynecology Clinic, Daito City, Osaka, Japan; 9 Department of Obstetrics and Gynecology, Hirakata City Hospital, Hirakata City, Osaka, Japan; 10 Department of Obstetrics and Gynecology, Iito Obstetrics and Gynecology Clinic, Kadoma City, Osaka, Japan; 11 Department of Obstetrics and Gynecology, Osamura Obstetrics and Gynecology Clinic, Yawata City, Kyoto, Japan; 12 Department of Obstetrics and Gynecology, Fujiwara Obstetrics and Gynecology Clinic, Katano City, Osaka, Japan; 13 Department of Obstetrics and Gynecology, Kayashima Ikuno Hospital, Kadoma City, Osaka, Japan; 14 Department of Obstetrics and Gynecology, Kamiya Obstetrics and Gynecology Clinic, Kadoma City, Osaka, Japan; 15 Department of Obstetrics and Gynecology, Kansai Medical University Medical Center, Moriguchi City, Osaka, Japan; University of Rwanda College of Medicine and Health Sciences, RWANDA

## Abstract

Many pregnant women worldwide use bicycles as a mode of transportation. However, there is limited evidence regarding attitudes toward and the safety of cycling as a mode of transportation during pregnancy. This study aimed to clarify the prevalence of cycling as a mode of transportation before and during pregnancy and the adverse events associated with cycling during pregnancy. An anonymous questionnaire survey was conducted among pregnant women who owned a bicycle, were at 37 weeks of gestation, and agreed to participate in the study at 14 institutions managing deliveries in Japan in 2024. Participants were provided with request forms containing a QR code linking to an anonymous questionnaire on Google Forms™. Among 1,130 women, the proportion who cycled for transportation decreased significantly from 80.8% before pregnancy to 51.2% during pregnancy (*P* < 0.001). One-third (34.2%) of women who cycled during pregnancy continued cycling until 37 weeks of gestation. The incidences of threatened abortion and preterm labor were similar regardless of cycling status during pregnancy. Of the 579 women who cycled during pregnancy, 21 (3.6%) fell off a bicycle, of whom 15 were injured. Eight women (1.4%) were involved in a bicycle accident during pregnancy, of whom four were injured. These adverse events occurred irrespective of gestational age. Logistic regression analysis showed that women who cycled ≥5 days per week during pregnancy had a significantly higher risk of falling off a bicycle (odds ratio, 3.17). Pregnancy was associated with a significant decrease in cycling as a mode of transportation; however, cycling-related adverse events during pregnancy were uncommon. Women who cycle ≥5 days per week during pregnancy should exercise particular caution to avoid falls. Safe cycling practices and helmet use should be more widely promoted during pregnancy.

## Introduction

Urban areas in developed countries typically offer multiple modes of public transportation, including trains, subways, buses, and taxis, and many families also own private vehicles. Among pregnant women who use motor vehicles for transportation, the incidence of associated traffic accidents ranges from 0.9% to 3.2% [1–4]. Many pregnant women worldwide also use bicycles as a mode of transportation for themselves, with or without their child(ren), for purposes such as commuting to work or school, shopping, and picking up or dropping off children, rather than for exercise or sport.

Previous studies have reported that the proportion of women who cycled for exercise decreased during pregnancy compared with before pregnancy [5–7]. Regarding modes of transportation during pregnancy, a previous study reported that, before 20 weeks of gestation, 66% of women used private transportation, 10% walked, 8% cycled, and 16% used public transportation (trains, buses, or taxis) to commute to work or school [8]. Another study reported that, during the third trimester, 53% of women drove a car, 32% used public transportation, and 15% walked or cycled to work [9].

Many pregnant women who cycle during pregnancy may be concerned about the risk of threatened abortion and/or threatened preterm labor. However, in general, few pregnant women who cycle experience abortion or preterm delivery. Nevertheless, many obstetricians are concerned about the risk of falls and bicycle accidents during pregnancy. In particular, as the maternal abdomen enlarges with advancing gestation, changes in body balance may increase the risk of falling while cycling. However, some pregnant women may need to continue cycling during pregnancy because their transportation options are limited to cycling or walking.

In recent years, approximately 130,000 injuries and 1,000 fatalities related to cycling have been reported annually in the United States, while approximately 70,000 cycling-related traffic accidents have been reported in Japan. However, neither the United States nor Japan reports data on the number of pregnant women involved in bicycle accidents during pregnancy. Limited data are available on cycling as a mode of transportation during pregnancy and the incidence of associated bicycle accidents. Furthermore, although cycling during pregnancy may be associated with risks such as threatened preterm labor and bicycle accidents, evidence regarding its prevalence and associated complications remains limited. Currently, there is insufficient evidence regarding attitudes toward and the safety of cycling as a mode of transportation during pregnancy.

This study aimed to clarify recommendations regarding cycling as a mode of transportation during pregnancy by investigating its prevalence and associated adverse events. The findings may provide evidence to inform pregnant women, healthcare professionals, and policymakers about safe cycling practices during pregnancy.

## Materials and methods

### Study design

An anonymous questionnaire survey was conducted.

### Setting

This study was conducted at 14 institutions in Japan that managed deliveries in 2024. The survey area comprised the North Kawachi secondary medical area in Osaka Prefecture (Hirakata, Neyagawa, Kadoma, Moriguchi, Daito, Shijonawate, and Katano cities; population, approximately 1.2 million), as well as Yawata City in Kyoto Prefecture, which is contiguous with Hirakata City. The participating institutions included one perinatal maternal center, four general hospitals with obstetrics departments, and nine maternity clinics with inpatient beds.

Within the survey area, almost all pregnant women with preterm delivery or severe, life-threatening injuries are transferred to Kansai Medical University Hospital in Hirakata City, which serves as both a perinatal maternal center and an emergency critical care center.

### Survey questions and response options

The questions and response options for the anonymous questionnaire survey were developed with reference to those used in previous studies investigating attitudes toward and patterns of driving or riding in motor vehicles and motor vehicle accidents during pregnancy in Japan [2,3]. All questions and response options were reviewed and edited for errors by a specialist in anonymous questionnaire surveys who was not an obstetrician (YM). Fourteen obstetricians (excluding YM) then completed the questionnaire and reviewed and revised its content. Finally, the final version of the anonymous questionnaire survey (S1 Table) was reviewed by the Kansai Medical University Hospital Institutional Review Board.

The questionnaire survey (S1 Table) consisted of 37 questions covering five themes: 1) basic information of participants, 2) cycling attitudes and habits before pregnancy, 3) those during pregnancy, 4) adverse events during pregnancy, and 5) opinions on helmet use for cycling, which recently became a “voluntary obligation “since April 1, 2023, in Japan.

### Distribution of survey requests

Pregnant women at 37 weeks of gestation at the participating institutions were provided with request forms containing a QR code that provided access to an anonymous questionnaire on Google Forms™. Participation was voluntary, and the survey was available from January 1 to December 31, 2024 (S1 Table).

### Informed consent

Informed consent was obtained through the first question of the survey (S1 Table):

Q1. Do you agree to participate in this questionnaire survey?

A1. 1) Yes. 2) No → Survey terminated.

All personal information was kept confidential.

### Inclusion and exclusion criteria

Women who owned a bicycle were eligible for inclusion, whereas those who did not own a bicycle were excluded. It was assumed that women who were unable to cycle would not own a bicycle. Women who owned a bicycle but did not use it were retained as the control group. Thus, all women who owned a bicycle were eligible for inclusion and were classified into two groups: (1) women who cycled and (2) women who did not cycle. Women who owned a bicycle but did not answer the questions regarding cycling before or during pregnancy were excluded because of missing data.

### Adverse events during pregnancy

Women who cycled during pregnancy were asked whether they had experienced bicycle accidents and/or falls from a bicycle, as well as threatened preterm labor and/or threatened abortion during pregnancy (S1 Table). In this study, women who reported having been diagnosed by an obstetrician with threatened preterm labor and/or threatened abortion because of a risk of preterm delivery or abortion were classified as having these conditions.

Women who reported an adverse event involving another party (a pedestrian, bicycle, motor vehicle, or other object) while cycling during pregnancy were classified as having experienced a “bicycle accident.” Women who reported falling from a bicycle without involvement of another party were classified as having experienced a “fall from a bicycle.” All reported bicycle accidents and falls from a bicycle were included regardless of whether the woman or any child riding with her sustained an injury.

### Primary and secondary outcomes

The primary outcome was the difference in attitudes toward and patterns of cycling as a mode of transportation before and during pregnancy. The secondary outcomes were the frequency of and risk factors for adverse events, including bicycle accidents, falls from a bicycle, threatened preterm labor, and threatened abortion, among women who cycled during pregnancy.

### Statistical analysis

Data are presented as numbers and frequencies. Statistical analyses were performed using JMP Pro, version 19.0 (SAS Institute Inc., Cary, NC, USA). Fisher’s exact test was used to analyze categorical variables. *P* values <0.05 were considered statistically significant. Multivariate logistic regression analysis was performed using variables with *P* < 0.05 in the univariate analysis.

### Ethics statement

This study was conducted in accordance with the principles of the Declaration of Helsinki and was approved by the Kansai Medical University Hospital Institutional Review Board (No. 2023254; December 13, 2023).

## Results

### Grouping of women enrolled in the survey

**Fig 1:**
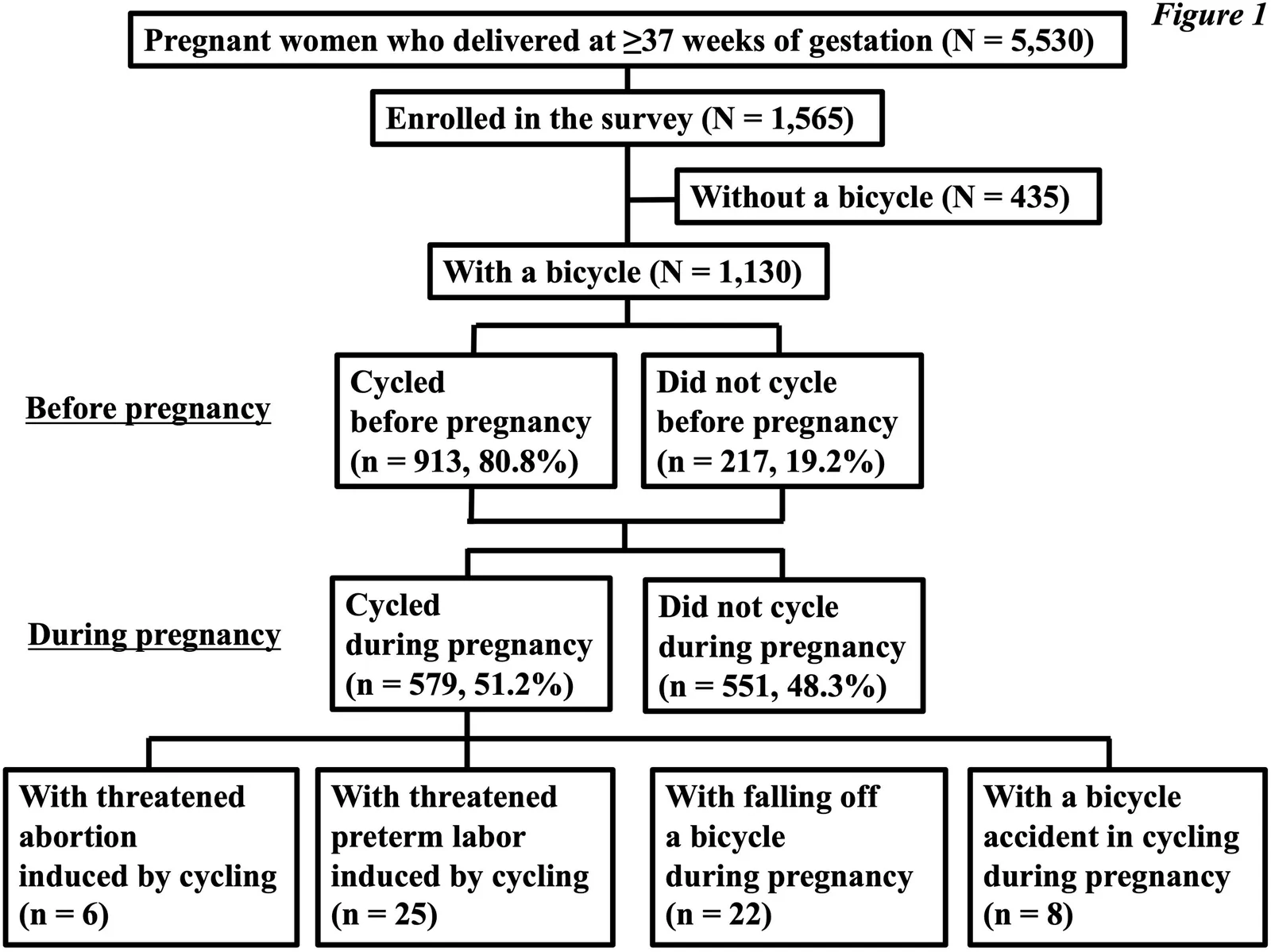
Flowchart of pregnant women enrolled in the survey.

Of the 5,530 women who delivered at ≥37 weeks of gestation across the 14 participating institutions, 1,565 (28.3%) were enrolled. Of these, 1,130 owned a bicycle and 435 did not. Therefore, 1,130 women (20.4% of the 5,530 women) were included in the study (Fig 1).

### Differences in cycling as a mode of transportation before and during pregnancy

Among the 1,130 women who owned bicycles, the proportion who cycled was significantly higher before pregnancy than during pregnancy (80.8% vs. 51.2%, *P* < 0.001). Multivariate analysis showed that the proportions of women who cycled every day (21.8% vs. 14.0%, *P* = 0.002), cycled ≥5 days per week (57.9% vs. 52.0%, *P* = 0.001), and primarily cycled for grocery shopping (30.9% vs. 23.3%, *P* = 0.013) were significantly lower during pregnancy than before pregnancy) (Table 1). Conversely, the proportion of women who cycled on sidewalks rather than roadways was significantly higher during pregnancy than before pregnancy (60.4% vs. 54.8%, *P* = 0.002).

**Table 1:** Participant characteristics and cycling patterns before and during pregnancy.

Characteristic		Women cycling before pregnancy (n = 913, 80.8%)	Women cycling during pregnancy (n = 579, 51.2%)	Univariate analysis, *P* value	Multivariate analysis, *P* value
**Participant characteristics**	Age ≥ 35 years	292 (32.0%)	201 (34.7%)	0.284	
	Multiparous	406 (44.5%)	188 (32.5%)	<0.001	NS
	Used a family vehicle	736 (80.6%)	473 (81.7%)	0.636	
	Used a helmet while cycling	113 (12.4%)	73 (12.6%)	0.936	
**Cycling characteristics**	Cycled every day	199 (21.8%)	81 (14.0%)	<0.001	0.002
	Cycled ≥5 days per week	529 (57.9%)	301 (52.0%)	0.025	0.001
	Cycled on sidewalks but not roadways	501 (54.8%)	350 (60.4%)	0.036	0.008
	Always wore a helmet while cycling	35 (3.8%)	27 (4.7%)	0.428	
	Cycled with children	433 (47.4%)	347 (59.9%)	<0.001	NS
	Children wore a helmet while riding on the bicycle	337 [77.8%, 337/433]	259 [74.6%, 259/347]	0.309	
	Primarily cycled to work	373 (40.9%)	196 (33.9%)	0.007	NS
	Primarily cycled to drop off and pick up children	231 (25.3%)	218 (37.7%)	<0.001	NS
	Primarily cycled for grocery shopping	282 (30.9%)	135 (23.3%)	0.002	0.013

Data are presented as number (percentage). NS, not significant.

### Cycling characteristics before and during pregnancy

Multivariate analysis revealed no significant differences in demographic or cycling characteristics between women who cycled before pregnancy (n = 913, 80.8%) and those who did not cycle before pregnancy (n = 217, 19.2%) (Table 2).

**Table 2:** Demographic and cycling characteristics during pregnancy according to cycling status before pregnancy.

Characteristic		Overall (n = 1,130)	Women who cycled before pregnancy (n = 913, 80.8%)	Women who did not cycle before pregnancy (n = 217, 19.2%)	Univariate analysis *P* value	Multivariate analysis *P* value
**Participant characteristics**	Age ≥ 35 years	342 (30.3%)	292 (32.0%)	50 (23.0%)	0.011	NS
	Multiparous	594 (52.6%)	507 (55.5%)	87 (40.1%)	<0.001	NS
	Used a family vehicle	922 (81.6%)	736 (80.6%)	186 (85.7%)	0.097	
	Used a helmet while cycling	134 (11.9%)	113 (12.4%)	21 (9.7%)	0.295	
	Cycled during pregnancy	579 (51.2%)	563 (61.7%)	16 (7.4%)	<0.001	NS
**Cycling characteristics during pregnancy**		**n = 579**	**n = 563**	**n = 16**	***P* value**	***P* value**
**Using cycling as a mode of transportation during pregnancy**	Cycled every day	81 (14.0%)	79 (14.0%)	2 (12.5%)	0.999	
	Cycled ≥5 days per week	301 (52.0%)	294 (52.2%)	7 (43.8%)	0.615	
	Cycled on sidewalks but not roadways	350 (60.7%)	343 (61.0%)	7 (46.7%)	0.291	
	Always wore a helmet while cycling	27 (4.7%)	25 (4.5%)	2 (12.5%)	0.169	
	Cycled with children	347 (61.4%)	338 (61.6%)	9 (56.3%)	0.795	
	Children wore a helmet while riding on the bicycle	259 (74.6%)	252 (72.6%)	7 (77.8%)	0.999	
	Primarily cycled to work	196 (33.9%)	193 (34.3%)	3 (18.8%)	0.285	
	Primarily cycled to drop off and pick up their children	218 (37.7%)	211 (37.5%)	7 (43.8%)	0.610	
	Primarily cycled for grocery shopping	135 (23.3%)	131 (23.3%)	4 (25.0%)	0.773	

Data are presented as number (percentage). NS, not significant.

Multivariate analysis showed that women who cycled during pregnancy (n = 579, 51.2%) were significantly more likely than those who did not cycle during pregnancy (n = 551, 48.8%) to have cycled ≥5 days per week before pregnancy (69.5% vs. 39.4%, *P* < 0.001) and to have cycled with children before pregnancy (63.9% vs. 23.6%, *P* < 0.001) (Table 3).

**Table 3:** Participant characteristics, adverse events during pregnancy, and pre-pregnancy cycling characteristics according to cycling status during pregnancy.

Characteristic		Overall (n = 1,130)	Women who cycled during pregnancy (n = 579, 51.2%)	Women who did not cycle during pregnancy (n = 551, 48.8%)	Univariate analysis, *P* value	Multivariate analysis, *P* value
**Participant characteristics**	Age ≥ 35 years	342 (30.3%)	201 (34.7%)	141 (25.6%)	<0.001	NS
	Multiparous	594 (52.6%)	391 (67.5%)	203 (36.8%)	<0.001	NS
	Used a family vehicle	922 (81.6%)	473 (81.7%)	449 (81.5%)	0.939	
	Used a helmet while cycling	134 (11.9%)	73 (12.6%)	61 (11.1%)	0.462	
	Cycled before pregnancy	913 (80.8%)	563 (97.2%)	350 (63.5%)	<0.001	NS
**Adverse events during pregnancy**	Threatened abortion	11 (1.0%)	6 (1.0%)	5 (0.9%)	0.999	
	Threatened preterm labor	51 (4.5%)	25 (4.3%)	26 (4.7%)	0.776	
	Fell off a bicycle	21 (1.9%)	21 (3.6%)	–	–	
	Involved in a bicycle accident	8 (0.7%)	8 (1.4%)	–	–	
**Cycled before pregnancy**		**n = 913**	**n = 563**	**n = 350**	***P* value**	***P* value**
**Cycling characteristics before pregnancy**	Cycled every day	199 (21.8%)	153 (27.2%)	46 (13.1%)	<0.001	NS
	Cycled ≥5 days per week	529 (57.9%)	391 (69.5%)	138 (39.4%)	<0.001	0.001
	Cycled on sidewalks but not roadways	501 (54.9%)	327 (58.1%)	174 (49.7%)	0.014	NS
	Always wore a helmet while cycling	35 (3.8%)	19 (3.4%)	16 (4.6%)	0.379	
	Cycled with children	433 (47.4%)	352 (63.9%)	81 (23.6%)	<0.001	<0.001
	Children wore a helmet while riding on the bicycle	337 (77.8%)	264 (75.0%)	73 (90.1%)	0.003	NS
	Primarily cycled to work	370 (40.5%)	238 (42.3%)	132 (37.7%)	0.188	
	Primarily cycled to drop off and pick up children	231 (25.3%)	189 (33.6%)	42 (12.0%)	<0.001	NS
	Primarily cycled for grocery shopping	282 (30.9%)	119 (21.1%)	163 (46.6%)	<0.001	NS

Data are presented as number (percentage). NS, not significant.

The incidences of threatened abortion and threatened preterm labor were comparable between women who cycled and those who did not cycle during pregnancy (1.0% vs. 0.9% and 4.3% vs. 4.7%, respectively).

One-third (34.2%) of women who cycled during pregnancy continued cycling until 37 weeks of gestation, whereas approximately one-fourth (25.9%) stopped cycling because they believed that cycling could adversely affect their pregnancy (Table 4).

**Table 4:** Patterns of cycling as a mode of transportation during pregnancy.

Characteristic	Overall	n = 579
**Gestational age at cessation of cycling (weeks)**	<19	95 (16.4%)
	20–27	114 (19.7%)
	28–36	172 (29.7%)
	≥37 (continued cycling at the time of survey participation)	198 (34.2%)
**Primary reason for stopping cycling**	Did not stop cycling (continued cycling at the time of survey participation)	198 (34.2%)
	Believed that cycling was not good for pregnancy	150 (25.9%)
	Pregnancy-related physical changes made cycling difficult	76 (13.1%)
	Took a leave of absence or left employment because of pregnancy	58 (10.0%)
	Family opposed cycling during pregnancy	42 (7.3%)
	Threatened preterm labor	7 (1.2%)
	Experienced a bicycle accident	2 (0.3%)
	Others	16 (2.8%)

Data are presented as number (percentage).

### Adverse events

Of the 579 women who cycled during pregnancy, 6 (1.0%) experienced threatened abortion and 25 (4.3%) experienced threatened preterm labor (Table 5). Of the 25 women who experienced threatened preterm labor, 20 (80.0%) required hospitalization. Moreover, 21 women (3.6%) fell off a bicycle during pregnancy. Of these, 15 (71.4%) sustained injuries, although none required hospitalization. Falls from a bicycle occurred across different gestational periods. Thirteen women (61.9%) fell while riding with their children, and 4 of these 13 children (30.8%) were injured. Eight women were involved in a bicycle accident during pregnancy, of whom 4 (50.0%) sustained injuries, although none required hospitalization. Bicycle accidents occurred across different gestational periods. Five women (62.5%) were involved in an accident while riding with their children, and 2 of the 8 children (25.0%) were injured. Of the eight bicycle accidents, 4 (50.0%) involved a motor vehicle, 3 (37.5%) involved another bicycle, and 1 (12.5%) involved a pedestrian.

**Table 5:** Adverse events among women who cycled as a mode of transportation during pregnancy.

Adverse event/characteristic	Women who cycled during pregnancy	n = 579
**Threatened abortion**	Overall	6 (1.0%)
	Required hospitalization	0/6 (0.0%)
**Threatened preterm labor**	Overall	25 (4.3%)
	Required hospitalization	20/25 (80.0%)
**Fell off a bicycle**	**Overall**	21 (3.6%)
	**Sustained an injury**	15/21 (71.4%)
	• Head and/or face	1/15 (6.7%)
	• Arm(s)	4/15 (26.7%)
	• Chest and/or abdomen	1/15 (6.7%)
	• Back and/or hip	3/15 (20.0%)
	• Leg(s)	12/15 (80.0%)
	**Required hospitalization**	0/21 (0.0%)
	***•*** Wore a helmet at the time of the fall	3/21 (14.3%)
	***•*** Riding with children at the time of the fall	13/21 (61.9%)
	• Child sustained an injury	4/13 (30.8%)*
	**Gestational age at the time of the fall (weeks)**	
	***•*** ≤ 19	6/21 (28.6%)
	***•*** 20–27	8/21 (38.1%)
	***•*** 28–36	7/21 (33.3%)
**Bicycle accident**	**Overall**	8 (1.4%)
	***•*** Involving a motor vehicle	4/8 (50.0%)
	***•*** Involving another bicycle	3/8 (37.5%)
	***•*** Involving a pedestrian	1/8 (12.5%)
	**Sustained an injury**	4/8 (50.0%)
	• Head and/or face	1/4 (25.0%)
	• Arm(s)	2/4 (50.0%)
	• Chest and/or abdomen	0/4 (0.0%)
	• Back and/or hip	0/4 (0.0%)
	• Leg(s)	2/4 (50.0%)
	**Required hospitalization**	0/8 (0.0%)
	***•*** Wore a helmet at the time of the accident	1/8 (12.5%)
	***•*** Riding with children at the time of the accident	5/8 (62.5%)
	• Child sustained an injury	2/5 (40.0%) †
	**Gestational age at the time of the accident (weeks)**	
	• ≤ 19	3/8 (37.5%)
	• 20–27	2/8 (25.0%)
	• 28–36	3/8 (37.5%)

Data are presented as number (percentage).

* Injuries among children: head and/or face, n = 2; arm(s), n = 1; leg(s), n = 1.

†Injuries among children: head and/or face, n = 1; leg(s), n = 1.

### Association among participant characteristics, adverse events, and cycling characteristics

Among the 579 women who cycled during pregnancy, no distinctive characteristics were identified among those who experienced threatened abortion (n = 6), threatened preterm labor (n = 25), or bicycle accidents (n = 8) compared with those who did not experience these adverse events. Women who fell off a bicycle (n = 21) were significantly more likely to have cycled with their children both before and during pregnancy than those who did not fall off a bicycle (n = 558) (Table 6). Logistic regression analysis showed that women who cycled ≥5 days per week during pregnancy had a significantly higher risk of falling from a bicycle than those who cycled less frequently (81.0% [17/21] vs. 50.9% [284/558]; odds ratio, 3.17; 95% confidence interval, 1.14–11.3; *P* = 0.043).

**Table 6:** Demographics and adverse events of women who cycled during pregnancy.

Characteristic		All women who cycled during pregnancy (n = 579)	Threatened abortion (n = 6, 1.0%)	Threatened preterm labor (n = 25, 4.3%)	Fell off a bicycle (n = 21, 3.6%)	Involved in a bicycle accident (n = 8, 1.4%)
**Participant characteristics**	Age ≥ 35 years	201 (34.7%)	1 (16.7%)	4 (16.0%)	7 (33.3%)	3 (37.5%)
	Multiparous	391 (67.5%)	5 (83.3%)	20 (80.0%)	20 (95.2%) ^*^	7 (87.5%)
	Used a family vehicle	472 (81.5%)	4 (66.7%)	22 (88.0%)	17 (90.0%)	8 (100%)
	Used a helmet while cycling	73 (12.6%)	0 (0.0%)	6 (24.0%)	5 (23.8%)	0 (0.0%)
**Before pregnancy**	Cycled	563 (97.2%)	5 (83.3%)	25 (100%)	20 (95.2%)	8 (100%)
	Cycled every day	153 (26.4%)	4 (66.7%)	13 (52.0%)	10 (47.6%)	4 (50.0%)
	Cycled ≥5 days per week	529 (91.4%)	5 (83.3%)	22 (88.0%)	20 (95.2%)	7 (87.5%)
	Cycled on sidewalks but not roadways	327 (56.5%)	3 (50.0%)	13 (52.0%)	13 (61.9%)	3 (37.5%)
	Cycled with children	352 (60.8%)	4 (66.7%)	20 (80.0%)	19 (90.4%) ^†^	7 (87.5%)
	Always wore a helmet while cycling	19 (3.3%)	0 (0.0%)	1 (4.0%)	0 (0.0%)	1 (12.5%)
**During pregnancy**	Cycled every day	81 (14.0%)	3 (50.0%)	3 (12.0%)	8 (38.1%)	2 (25.0%)
	Cycled ≥5 days per week	301 (52.0%)	4 (66.7%)	16 (64.0%)	17 (81.0%) ^‡^	6 (75.0%)
	Cycled on sidewalks but not roadways	350 (60.4%)	5 (83.3%)	14 (56.0%)	15 (71.4%)	4 (50.0%)
	Cycled with children	347 (59.9%)	4 (66.7%)	19 (76.0%)	20 (95.2%) ^§^	7 (87.5%)
	Always wore a helmet	27 (4.7%)	0 (0.0%)	2 (8.0%)	1 (4.8%)	0 (0.0%)

Data are presented as number (percentage).

* *P* = 0.004; † *P* = 0.005; ‡ *P* = 0.007; § *P* < 0.001 versus women who did not experience the respective adverse event among all women who cycled during pregnancy.

In multivariate logistic regression analysis, women who cycled ≥5 days per week during pregnancy had a significantly higher risk of falling off a bicycle than those who cycled less frequently (81.0% [17/21] vs. 50.9% [284/558]; odds ratio, 3.17; 95% confidence interval, 1.14–11.3; *P* = 0.043). Variables with *P* < 0.05 in the univariate analysis were included in the multivariate model.

## Discussion

This study describes the demographic and cycling characteristics of women who used bicycles as a mode of transportation during pregnancy, as well as the frequency of adverse events, including bicycle accidents, falling off a bicycle, threatened abortion, and threatened preterm labor.

In Japan, most women who cycle during pregnancy do so as a mode of transportation rather than as a form of exercise. Our survey revealed that the proportion of women who cycled for transportation decreased significantly from 80.8% before pregnancy to 51.2% during pregnancy. A study involving 575 primiparous women in Norway between 2009 and 2013 showed a significant increase in the use of private vehicles for transportation to work or school, from 58% before pregnancy to 64% in early pregnancy (*P* = 0.001), whereas the proportion who cycled decreased from 11% to 5% (*P* < 0.001) [8]. In our study, the main reason for discontinuing cycling as a mode of transportation during pregnancy was the belief that cycling was not good for pregnancy. Thus, women who were more cautious about their behavior during pregnancy, including cycling, may have been more likely to participate in this study.

Cycling as a mode of transportation is considered a form of “active transport,” similar to walking as a mode of transportation [10]. In previous studies not specifically involving pregnant women, physical activity, road transport–related injuries, and air pollution have been considered relevant exposures associated with active transport (cycling or walking plus cycling) [10]. According to the American College of Obstetricians and Gynecologists, safe physical activity during pregnancy is desirable for women without obstetric or medical complications or contraindications, and pregnant women should be encouraged to continue or initiate safe physical activities [11]. A previous review identified various barriers to physical activity (i.e., exercise) among pregnant women, including pregnancy-related symptoms, lack of time, limited access to childcare, and safety concerns. Conversely, facilitating factors included positive psychological experiences, family influence, and advice from healthcare professionals (including obstetricians) [12]. The facilitators and barriers associated with cycling as a mode of transportation may be similar to those associated with cycling as exercise during pregnancy. However, cycling for transportation additionally requires consideration of the risks of falls and traffic accidents. A previous expert review noted that controlled physical activity is safe during pregnancy and may reduce excessive gestational weight gain and associated pregnancy-related complications [13]. Further research is needed to determine the optimal frequency and duration of specific types of physical activity during pregnancy. Another review reported that the domains of physical activity most frequently assessed during pregnancy included occupational activity, housework, caregiving, sport/exercise, active living, and habitual activity [14]. However, few studies have examined attitudes toward and the safety of cycling specifically as a mode of transportation during pregnancy. Therefore, further research is needed to evaluate the benefits and risks of cycling as a mode of transportation during pregnancy.

Our study focused on four main adverse events potentially associated with cycling during pregnancy: threatened abortion, threatened preterm labor, falling off a bicycle, and bicycle accidents. However, the incidences of these events among women who cycled during pregnancy were low (1.0%–4.3%). The guidelines of the Japan Society of Obstetrics and Gynecology and the Japan Association of Obstetricians and Gynecologists do not specifically address the benefits and risks of cycling as a mode of transportation during pregnancy [15]. Nevertheless, aerobic exercise using a stationary bicycle is recommended as a preferred form of exercise during pregnancy (recommendation level B), as exercise does not increase maternal morbidity, including preterm birth (recommendation level B). However, pregnant women who develop threatened preterm labor are advised not to initiate or continue exercise or sports (recommendation level A). Women who develop symptoms suggestive of threatened preterm labor, such as abdominal tightness, lower abdominal pressure, uterine contractions, or vaginal bleeding, during exercise should consult an obstetrician (recommendation level B) [15]. However, current obstetric guidelines do not provide recommendations regarding cycling as a mode of transportation during pregnancy because of the limited evidence available.

In our study, women who cycled frequently during pregnancy had a significantly higher risk of falling off a bicycle but not of threatened preterm labor. In the United States, a prospective study involving 17 women during the last 8 weeks of pregnancy found that exercise using a stationary bicycle did not increase uterine activity [16]. In the Danish National Birth Cohort study [17,18] and the Norwegian Mother and Child Cohort Study [19], women who cycled as a form of physical activity during early and mid-pregnancy, respectively, had a slightly lower risk of preterm birth than those who did not engage in such activity. Thus, cycling as a mode of transportation during pregnancy may not necessarily increase the risk of preterm labor. In Japan, the overall rate of preterm delivery was 5.7% in 2023 [20]. In comparison, the incidence of threatened preterm labor among women who cycled during pregnancy was 4.3% (25/579) in our survey. However, our survey may not have captured women who experienced preterm delivery and/or were involved in a bicycle accident before the survey was administered at 37 weeks of gestation. Notably, within the survey area, almost all pregnant women with preterm delivery and/or severe, life-threatening injuries are transferred to Kansai Medical University Hospital in Hirakata City, which has a perinatal maternal center and an emergency critical care center. Nevertheless, no cycling-related cases were treated at the university hospital in 2024. The incidence of bicycle accidents during pregnancy in our study (1.4%) was within the range of motor vehicle accident rates reported in previous studies (0.9%–3.2%) [1–4].

In Japan, the proportion of bicycle accidents among all traffic accidents has increased, reaching its highest level in the past 20 years in 2023 (23.5%), with approximately 40% of bicycle accidents occurring on sidewalks [21]. Furthermore, approximately 50% of cyclists who died in bicycle accidents sustained head injuries [22]. In our survey, however, only 1 (12.5%) of the 8 women involved in a bicycle accident during pregnancy sustained an injury to the head and/or face.

Following the enforcement of the revised Japanese Road Traffic Act on April 1, 2023, helmet use has been recommended for all cyclists in Japan [21]. The Act recommends the following: (1) cyclists should make reasonable efforts to wear a helmet; (2) when carrying a child on a bicycle, cyclists should make reasonable efforts to ensure that the child wears a helmet; and (3) parents and guardians should make reasonable efforts to ensure that their children wear a helmet when cycling [23]. According to surveys conducted by the National Police Agency in July 2024 [24] and July 2025 [25], the proportions of cyclists wearing helmets nationwide were 17.0% and 21.2%, respectively, whereas the corresponding proportions in Osaka Prefecture were 5.5% and 7.2%, respectively. In our study, conducted in part of Osaka Prefecture in 2024, the proportions of women who always wore a helmet while cycling before and during pregnancy (3.8% and 4.7%, respectively) were similarly low. Therefore, helmet use while cycling should be actively encouraged among women, regardless of pregnancy status.

Previous studies on motor vehicle accidents and seatbelt use during pregnancy have suggested that greater awareness of driving safety among pregnant women may be associated with a reduced risk of motor vehicle accidents [2] and that educational leaflets on seatbelt use can increase consistent seatbelt use during pregnancy [3]. Accordingly, seatbelt use should be more widely promoted among pregnant women to reduce morbidity associated with motor vehicle accidents during pregnancy [2,3]. Similarly, wearing a helmet while cycling can reduce the risk of head injury and death among cyclists. In our study, only 14.3% of the 21 women who fell off a bicycle and 12.5% of the 8 women involved in a bicycle accident were wearing a helmet at the time of the event (Table 5). In Japan, there is no national certification scheme for cycling as a mode of transportation. Therefore, government authorities and obstetricians should more actively promote safe cycling practices and helmet use among pregnant women to reduce morbidity associated with falls and bicycle accidents while cycling as a mode of transportation during pregnancy.

This study has four limitations. First, the survey had a low response rate. Second, women who delivered at <37 weeks of gestation were not included, potentially resulting in the omission of adverse events related to cycling that occurred earlier in pregnancy. Third, the number of questions was limited to facilitate participation in the anonymous questionnaire survey. Consequently, we were unable to collect and examine detailed information on respondents’ socioeconomic characteristics (educational background, occupation, income, etc.). Fourth, there are concerns regarding the reliability and validity of the questionnaire. The final version of the anonymous questionnaire (S1 Table) had a Cronbach’s α of 0.652 in this study, indicating moderate but not high internal consistency. Although the survey questions and response options were developed with careful consideration of content validity, a pilot study involving pregnant women was not conducted. Therefore, the validity of the final questionnaire (S1 Table) could not be fully established.

## Conclusion

The proportion of women who used cycling as a mode of transportation decreased significantly from before to during pregnancy (from approximately 80% to 50%). The incidences of falls from a bicycle and bicycle accidents during pregnancy were low in our survey. However, women who cycle ≥5 days per week during pregnancy should exercise particular caution to avoid falls. Safe cycling practices and helmet use should be more widely promoted among pregnant women to reduce morbidity associated with falls and bicycle accidents while cycling as a mode of transportation during pregnancy.

## Supporting information

S1 Table Anonymous questionnaire survey on cycling during pregnancy among women at 37 weeks of gestation. (DOCX)

S1 Dataset Dataset of anonymous questionnaire survey generated and analyzed in this study. (XLSX)
