# Supplementary material for: Using cycling as transportation and related adverse events during pregnancy: An anonymous questionnaire survey in urban Japan
Source: PLoS One. 2026 Sep 22;21(9):e0358856. doi: 10.1371/journal.pone.0358856 (PMC13596788; doi:10.1371/journal.pone.0358856)
Supplement: Supplementary file 1 [file pone.0358856.s001.docx]

**S1 Table.** The anonymous questionnaire survey on cycling during pregnancy for pregnant women at 37 weeks of gestation

**Q1. Do you agree to participate in this questionnaire survey?**

A1. 1) Yes.　2) No → Finished.

**Q2. How old are you (years)?**

A2. 1) ≤19.　2) 20–24. 3) 25–29. 4) 30–34. 5) 35–39. 6) ≥40.

**Q3. How many children do you have?**

A3. 1) None (primipara).　2) One. 3) Two. 4) Three or more.

**Q4. Do you own a car?**

A4. 1) Yes,　2) No.

**Q5. Do you own a bicycle?**

A5. 1) Yes.　2) No → Finished.

**Q6. Do you own a helmet for cycling as transportation?**

A6. 1) Yes.　2) No.

**Q7. Did you cycle before pregnancy?**

A7. 1) No → Please go to Q13. 2) Yes → Please go to Q8.

**Q8. How often did you cycle before pregnancy?**

A8. 1) Every day. 2) 5–6 days per week. 3) 3–4 days per week. 4) 1–2 days per week.

**Q9. What was your main purpose for cycling before pregnancy?**

A9. 1) Commuting to work or school. 2) Shopping. 3) Picking up and dropping off children. 4) Exercise. 5) Other (Please explain specifically: ).

**Q10. Where did you use cycling as a transportation before pregnancy?**

A10. 1) Mainly on the roadway. 2) Mainly on the sidewalk.

**Q11. Did you wear a helmet when cycling before pregnancy?**

A11. 1) Every time. 2) Sometimes. 3) Never.

**Q12. Did you ever let your children ride with you when cycling before pregnancy?**

A12. 1) No. 2) Yes (one child with a helmet for cycling). 3) Yes (one child without a helmet for cycling). 4) Yes (two or more children with helmets for cycling). 5) Yes (two or more children without helmets for cycling).

**Q13. Did you cycle during pregnancy?**

A13. 1) No → Please go to Q34. 2) Yes → Please go to Q14.

**Q14. When did you stop cycling during pregnancy?**

A14. 1) ≤19 weeks of gestation. 2) 20–27 weeks of gestation. 3) 28–36 weeks of gestation. 4) ≥37 weeks of gestation (still cycling).

**Q15. How often did you cycle during pregnancy?**

A15. 1) Every day. 2) 5–6 days per week. 3) 3–4 days per week. 4) 1–2 days per week.

**Q16. What was your main purpose for cycling during pregnancy?**

A16. 1) Commuting to work or school. 2) Shopping. 3) Picking up and dropping off children. 4) Exercise. 5) Other (Please explain specifically: ).

**Q17. Where did you cycle during pregnancy?**

A17. 1) Mainly on the roadway. 2) Mainly on the sidewalk.

**Q18. Did you wear a helmet for cycling during pregnancy?**

A18. 1) Every time. 2) Sometimes. 3) Never.

**Q19. Did you ever let your children ride with you when cycling during pregnancy?**

A19. 1) No. 2) Yes (one child with a helmet for cycling). 3) Yes (one child without a helmet for cycling). 4) Yes (two or more children with helmets for cycling). 5) Yes (two or more children without helmets for cycling).

**Q20. Have you ever fallen off a bicycle while cycling during pregnancy?**

A20. 1) No → Please go to Q27. 2) Yes (1 time) → Please go to Q21. 3) Yes (2 times or more) → Please go to Q21.

**Q21. When did you fall off a bicycle while cycling during pregnancy?**

A21. 1) ≤19 weeks of gestation. 2) 20–27 weeks of gestation. 3) 28–36 weeks of gestation. 4) ≥37 weeks of gestation.

**Q22. Did you wear a helmet for cycling when you fell off a bicycle during pregnancy?**

A22. 1) Yes. 2) No.

**Q23. Where did you bruise when you fell off a bicycle during pregnancy?**

A23. 1) Nowhere. 2) Head or face. 3) Chest. 4) Abdomen. 5) Hip or pelvis. 6) Arm(s). 7) Leg(s). 8) Other (Please explain specifically: ).

**Q24. Were you hospitalized when you fell off your bicycle during pregnancy?**

A24. 1) No. 2) Yes.

**Q25. Were your children riding with you when you fell off a bicycle during pregnancy?**

A25. 1) No → Please go to Q27. 2) Yes (one child with a helmet for cycling) → Please go to Q26. 3) Yes (one child without a helmet for cycling) → Please go to Q26. 4) Yes (two or more children with helmets for cycling) → Please go to Q26. 5) Yes (two or more children without helmets for cycling) → Please go to Q26.

**Q26. Where did your children bruise when you fell off a bicycle during pregnancy?**

A26. 1) Nowhere. 2) Head or face. 3) Chest. 4) Abdomen. 5) Hip or pelvis. 6) Arm(s). 7) Leg(s). 8) Other (Please explain specifically: ).

**Q27. Have you ever had a bicycle accident while cycling during pregnancy?**

A27. 1) No → Please go to Q28. 2) Yes (The other party in the accident was a human.). 3) Yes (The other party in the accident was a bicycle.). 4) Yes (The other party in the accident was a vehicle.). 5) Yes. (The other party in the accident was something else. → Please explain specifically: ).

**Q28. When did you have a bicycle accident(s) while cycling during pregnancy?**

A28. 1) ≤19 weeks of gestation. 2) 20–27 weeks of gestation. 3) 28–36 weeks of gestation. 4) ≥37 weeks of gestation.

**Q29. Did you wear a helmet for cycling when you had your accident(s) during pregnancy?**

A29. 1) Yes. 2) No.

**Q30. Where did you bruise when you had an accident(s) while cycling during pregnancy?**

A30. 1) Nowhere. 2) Head or face. 3) Chest. 4) Abdomen. 5) Hip or pelvis. 6) Arm(s). 7) Leg(s). 8) Other (Please explain specifically: ).

**Q31. Were you hospitalized when you had a bicycle accident during pregnancy?**

A31. 1) No. 2) Yes.

**Q32. Did you ever let your children ride with you when you had a bicycle accident(s) during pregnancy?**

A32. 1) No → Please go to Q34. 2) Yes (one child with a helmet for cycling) → Please go to Q33. 3) Yes (one child without a helmet for cycling) → Please go to Q33. 4) Yes (two or more children with helmets for cycling) → Please go to Q33. 5) Yes (two or more children without helmets for cycling) → Please go to Q33.

**Q33. Where did your children bruise when you had your bicycle accident(s) during pregnancy?**

A33. 1) Nowhere. 2) Head or face. 3) Chest. 4) Abdomen. 5) Hip or pelvis. 6) Arm(s). 7) Leg(s). 8) Other (Please explain specifically: ).

**Q34. Have you ever had a threatened abortion or threatened preterm labor during pregnancy?**

A34. 1) No. 2) Threatened abortion with treatment at home. 3) Threatened abortion with hospitalization. 4) Threatened preterm labor with treatment at home. 5) Threatened preterm labor with hospitalization.

**Q35. What was the main reason you refused/stopped cycling during pregnancy?**

A35. 1) You thought cycling was not good for the pregnancy. 2) Your family opposed riding a bicycle. 3) Your physical changes induced by pregnancy made cycling difficult. 4) You quit your job or took a leave of absence due to pregnancy, so you did not need to cycle. 5) You had a bicycle accident while cycling (before/ during pregnancy). 6) You hadn’t cycled before pregnancy. 7) Other (Please explain specifically: ). 8) You continued cycling.

**Q36. Did you know that wearing a helmet for cycling became a “voluntary obligation” since April 1, 2023, in Japan?**

A36. 1) Yes. 2) No.

**Q37. Did you make an effort to wear a helmet for cycling since wearing a helmet for cycling became a “voluntary obligation”?**

A37. 1) Yes (You have worn it before becoming the “voluntary obligation”). 2) Yes (You have started wearing a helmet for cycling). 3) Yes (You thought to start wearing a helmet for cycling). 4) No (You did not think to start wearing a helmet for cycling).
